# Rore: robust and efficient antioxidant protein classification via a novel dimensionality reduction strategy based on learning of fewer features

**DOI:** 10.1186/s44342-024-00026-z

**Published:** 2024-12-04

**Authors:** Chaolu Meng, Yongqi Hou, Quan Zou, Lei Shi, Xi Su, Ying Ju

**Affiliations:** 1 College of Computer and Information Engineering, Inner Mongolia Agricultural University, Hohhot, China; 2 Inner Mongolia Autonomous Region Key Laboratory of Big Data Research and Application of Agriculture and Animal Husbandry, Hohhot, China; 3 School of Computer Science, Inner Mongolia University, Hohhot, China; 4 Institute of Fundamental and Frontier Sciences, University of Electronic Science and Technology of China, Chengdu, China; 5 Department of Spine Surgery, Changzheng Hospital, Naval Medical University, Huangpu District, No. 415, Fengyang Road, Shanghai, China; 6 Foshan Women and Children Hospital, Foshan, China; 7 School of Informatics, Xiamen University, Xiamen, China

**Keywords:** Dimensionality reduction, Robust feature, Protein classifier, Protein sequence

## Abstract

In protein identification, researchers increasingly aim to achieve efficient classification using fewer features. While many feature selection methods effectively reduce the number of model features, they often cause information loss caused by merely selecting or discarding features, which limits classifier performance. To address this issue, we present Rore, an algorithm based on a feature-dimensionality reduction strategy. By mapping the original features to a latent space, Rore retains all relevant feature information while using fewer representations of the latent features. This approach significantly preserves the original information and overcomes the information loss problem associated with previous feature selection. Through extensive experimental validation and analysis, Rore demonstrated excellent performance on an antioxidant protein dataset, achieving an accuracy of 95.88% and MCC of 91.78%, using vectors including only 15 features. The Rore algorithm is available online at http://112.124.26.17:8021/Rore.

## Introduction

Antioxidant proteins produced by the human body can resist free radical damage. Identifying which human proteins are antioxidant can help prevent diseases such as cancer and cardiovascular disease [1–4]. For this purpose, machine learning models can be used. Model’s fewer features can improve the interpretability of the model and help researchers understand the underlying biological mechanisms [5–13]. Antioxidant protein identification based on machine learning has been performed in the past [14–17]. In 2016, Feng et al. developed the AodPred model based on optimal 3-gap dipeptides for feature selection to obtain 158-dimensional features for classification [18]. In 2020, Chun et al. identified 9808-dimensional hybrid features using 188D, N-gram, ACC-PSSM, and g-gap. The authors performed feature selection using MRMD, t-SNE, and optimal feature set selection methods and classified the resulting feature vectors using the random forest algorithm [19]. In 2023, Chao et al. used MRMD and dynamic programming to select of 473D feature vectors and to obtain 17-dimensional feature vectors for classification [20]. In general, the methods used in these studies use feature selection to reduce the antioxidant protein dimensions, which inevitably leads to information loss. Most importantly, potential relationships between the features are ignored, limiting the performance of the classifiers. In addition, unbalanced datasets for antioxidant protein identification lead to low MCC values, which indicates poor model performance in predicting a small number of classes [21–24]. Notably, in clinical applications, misdiagnoses caused by targeting only few classes may have fatal consequences [25–29]. To address this problem, we propose the Rore model, which achieves superior MCC values based on a feature dimensionality reduction algorithm and also preserves the original information and relationships between the features. The usage of a SMOTE algorithm that rebalances the dataset. Specifically, the Rore model was found to achieve 95.88% accuracy and a 91.78% MCC value using only 15-dimensional features. Our findings demonstrated Rore shows significant performance with fewer features.

## Methods

**Fig. 1:**
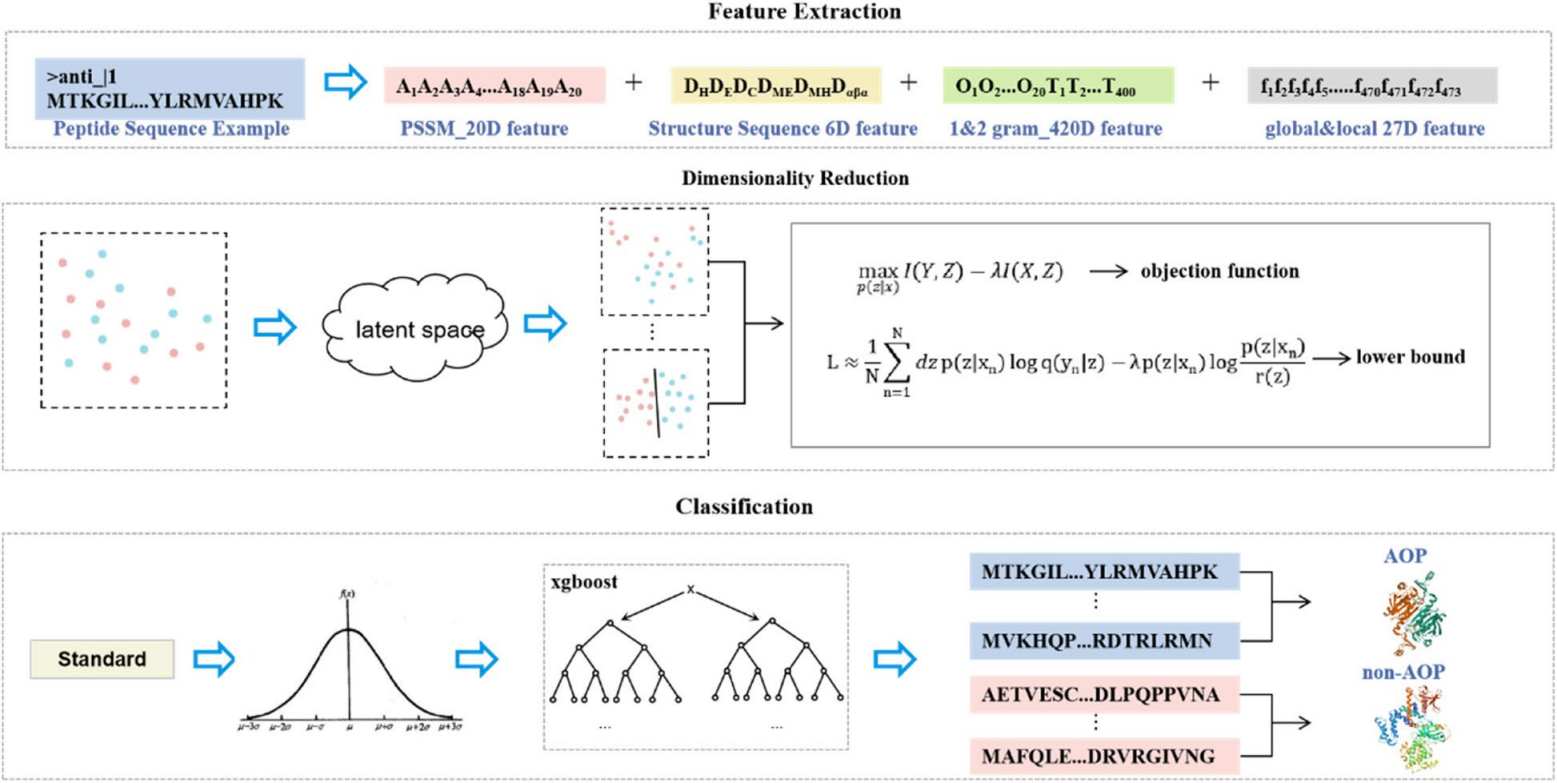
Overall structure of the Rore classifier. The Rore model is built in three steps: extracting 473-dimensional feature vectors from protein sequences, using the feature extraction method proposed by Wei, and selecting the most informative vectors among these according to the VFC feature dimensionality reduction method. Finally, the model is trained using the XGBoost algorithm

To address the problem of information loss, we developed a classification method based on a new feature-dimensionality reduction strategy called Rore. For this purpose, we first constructed an antioxidant protein dataset containing 3104 samples by screening and rebalancing data in the UniProt database using the SMOTE algorithm. Subsequently, a 473-dimensional feature vector was extracted (Fig. 1). To reduce feature dimensionality and maximize the retention of the original feature information, we propose a feature dimensionality reduction method, the variational feature compressor (VFC). This method is based on the idea of an information bottleneck. Through VFC processing, we obtain a final 15-dimensional feature vector. Finally, this 15-dimensional feature vector was fed to the XGBoost algorithm for classification.

### Benchmark dataset

We used the dataset collected in previous studies to allow a fair and comprehensive performance comparison with existing methods [18, 30, 31]. This dataset is unbalanced. The positive sample consists of sequences labeled as “antioxidant” from the UniProt database [32]. Samples containing “B,” “X,” “Z,” “O,” “U,” and “J” were eliminated due to uncertainty regarding their meanings [33], and only the sequences labeled “review” were retained for experimental validation. Negative samples were obtained from the PDB database using the PISCES procedure with an identification rate of no more than 20% (values less than 20%). The resulting dataset contained 1805 protein sequences, with 253 positive and 1552 negative samples.

$$\text{Data}={\text{positive}}_{+}+{\text{negative}}_{-}$$

Unbalanced datasets can lead to overfitting when training models. Thus, we rebalanced the dataset using the positive sample oversampling method, SMOTE [34], to obtain a 1:1 balanced dataset. The algorithm randomly selects a similarity point from the M similarity points closest to the sample point [35], and a new data point is generated through a linear link between the sample point and the randomly selected similarity point. After processing using the SMOTE algorithm, a dataset containing 3104 samples was obtained.

### Feature extraction

We used the feature extraction method proposed by Wei et al. [36]. This method uses PSI-BLAST [37] and PSI-PRED [38] algorithms to extract features. These two algorithms obtain feature information from the sequence and structural points of view [39–41]. We reasoned that the complementarity of these two types of features can improve the prediction accuracy. The specific steps were as follows:

The PSI-BLAST algorithm was first used to obtain a position-specific score matrix. Twenty feature vectors are obtained from the job-specific score matrix. The position-specific score matrix can be expressed as follows:

$${S}_{pssm}=\left[\begin{array}{ccc}{pse}_{\text{1,1}}& \cdots & {pse}_{\text{1,20}}\\ {pse}_{\text{2,1}}& \cdots & {pse}_{\text{2,20}}\\ \vdots & \ddots & \vdots \\ {pse}_{\text{20,1}}& \cdots & {pse}_{\text{20,20}}\end{array}\right]$$

where the score for mutation of residues at position i in the protein sequence S to j-type residues is denoted as ${pse}_{i,j}$. The 20 feature vectors to be extracted were the mean values ${D}_{pssm}$ of the mutated residues based on the 20 different types of mutated residues obtained during the evolutionary process [42], and ${D}_{pssm}$ can be denoted as follows:

$${D}_{pssm}=\left\{\overline{{A }_{j}}=\frac{1}{L}\sum_{i=1}^{L}{A}_{ij};1\le j\le 20\right\}$$

where ${A}_{j}$ denotes the mean value of the mutations occurring in the jth residue type during evolution.

In order to extract features from sequences containing richer evolutionary information, it is first necessary to transform each ${A}_{i,j}$ into a consensus sequence ${A}_{i,j}{\prime}$ with the following transformation formula:

$${A}_{ij}{\prime}={2}^{{A}_{ij}\times {BF}_{j}}$$

where ${BF}_{j}$ is obtained by dividing the number of amino acids by the number of sequences for all sequences in the PDB25 database [43]. Then, only a maximum value remains each row, and the new sequence ${A}_{con}$ obtained is the consensus sequence containing evolutionary information.

Next, 20 and 400 features were extracted from the consensus sequence using 1-g and 2-g algorithms, respectively [44]. The feature extraction using the 1-g and 2-g algorithms can be formulated as follows:

$${D}_{1-gram}=\left\{\frac{O\left({P}_{i}\right)}{L};1\le i\le 20\right\}$$

$${D}_{2-gram}=\left\{\frac{O\left({P}_{i}{P}_{j}\right)}{L};1\le i\le \text{20,1}\le j\le 20\right\}$$

where ${P}_{i}$ denotes the residue i, $\text{O}\left({P}_{i}\right)$ is the frequency of occurrence of the residue, and $\text{O}\left({P}_{i}{P}_{j}\right)$ denotes the frequency of occurrence of residue pairs. Using a weighted combination of the 1-g and 2-g algorithms [45, 46], 420 features ${D}_{con}$ were obtained, which can be denoted as follows:

$${D}_{con}=\left\{\frac{20{D}_{1-gram}}{420},\frac{400{D}_{2-gram}}{420}\right\}$$

PSI-PRED algorithm allows obtaining sequences and matrices of information about the structure from which features can be extracted, with 6 features for the former and 27 features for the latter. Where the secondary structure sequence is noted as ${A}_{str}={S}_{1}{S}_{2}{S}_{3}\cdots {S}_{L}\left(S\in \left\{H,E,C\right\}\right)$, H, E, and C denote the three states. The six features extracted from the sequence of relevant structural information obtained using the PSI-PRED algorithm were as follows:

$${D}_{H}=\frac{\sum_{i=1}^{{total}_{H}}{I}_{{H}_{i}}}{L\left(L-1\right)}$$

$${D}_{E}=\frac{\sum_{i=1}^{{total}_{E}}{I}_{{E}_{i}}}{L\left(L-1\right)}$$

$${D}_{C}=\frac{\sum_{i=1}^{{total}_{C}}{I}_{{C}_{i}}}{L\left(L-1\right)}$$

$${D}_{ME}=\frac{maxE}{L}$$

$${D}_{MH}=\frac{maxH}{L}$$

where ${total}_{H}$, ${total}_{E}$, and ${total}_{C}$ are the sums of ${A}_{str}$ in three states and ${I}_{H}$, ${I}_{E}$, and ${I}_{C}$ are the location indices of the three states. maxE and maxH represent the maximum continuous lengths of the two states in space. ${D}_{ME}$ and ${D}_{MH}$ are the normalized maximum lengths. Replacing consecutive helices in the secondary structure sequence with *α* and consecutive strands with *β*, ignoring coiled coils, results in a set of fragment sequences consisting of *α* and *β*. Based on the difference in the *α* and *β* arrangement in α/β proteins and α + β proteins, the two proteins can be distinguished by the following features:

$${D}_{\alpha \beta \alpha }=\frac{{total}_{\alpha \beta \alpha }}{L-2}$$

where $total_{{\beta}{\alpha}{\beta}}$ is the total number of occurrences of βαβ segments in the segmentation sequence ${A}_{str}$.

From the structural correlation matrix, we have extracted 27 global structural features. Local structural features were extracted from the structural correlation matrix. Matrix consists of L rows and three columns, with each column representing the three states. The structural probability matrix ${SPM}_{pro}$ can be expressed as follows:

$${SPM}_{pro}=\left[\begin{array}{ccc}{p}_{1,C}& {p}_{1,H}& {p}_{1,E}\\ {p}_{2,C}& {p}_{2,H}& {p}_{2,E}\\ \vdots & \vdots & \vdots \\ {p}_{L,C}& {p}_{L,H}& {p}_{L,E}\end{array}\right]$$

where $p_{i,C}\,p_{i,H}\,p_{i,E}$ are the probabilities of states C, H, and E, respectively. L is the size of the protein sequence. Based on the structure probability matrix, 3 and 24 features can be obtained from the global and local perspectives, respectively, where the global structural features can be denoted as follows:

$${D}_{global\_C}=\frac{1}{L}\sum_{i=1}^{L}{p}_{i,C}$$

$${D}_{global\_H}=\frac{1}{L}\sum_{i=1}^{L}{p}_{i,H}$$

$${D}_{global\_E}=\frac{1}{L}\sum_{i=1}^{L}{p}_{i,E}$$

The local structural features were obtained by dividing the structural correlation matrix obtained according to the PSI-PRED algorithm into eight submatrices by row, and each submatrix consisted of three columns. The computation of the features for each submatrix is consistent with the computation of the global structural features. We obtained the following eight local structural features:

$${D}_{local}=\left\{{D}_{local\_1C},{D}_{local\_1H},{D}_{local\_1E},\cdots ,{D}_{local\_8C}{D}_{local\_8H}{D}_{local\_8E}\right\}$$

where ${D}_{local\_iC},{D}_{local\_iH},{D}_{local\_iE}$ denote the probabilities of the submatrix in the three states; that is, 24 local structural features are obtained from the structural probability matrix.

In conclusion, the PSI-BLAST algorithm obtained 440 features, including 20 features, using a location-specific score matrix, and 420 features based on a weighted combination of 1-g and 2-g algorithms. In addition, PSI-PRED extracted 33 features, including 6 features based on the secondary structure sequence and 3 and 24 features based on the structural probability matrix from global and local perspectives, respectively. The total number of features was set to 473.

In conclusion, the PSI-BLAST algorithm obtained altogether 440 features, including 20 features by means of a location-specific score matrix and 420 features based on a weighted combination of 1-g and 2-g algorithms. In addition, PSI-PRED extracted 33 features, including 6 features based on the secondary structure sequence, and 3 features and 24 features based on the structural probability matrix from global and local perspectives, respectively. The total number of features is 473.

### Variational feature compressor

To make the variational information bottleneck [47] (VIB) available for improved feature dimensionality reduction, we propose a variational feature compressor (VFC). In order to extract from the 473D feature vectors all possible representations of nonlinear interaction effects between features, it is necessary to convert the 473D feature vectors into more compact nonlinear latent variables [48]. The conversion process can be represented as follows:

$${F}_{potential}=RELU\left(Dense\left({F}_{473D}\right)\right)$$

where ${F}_{473D}$ denotes the 473D feature vector obtained from 473D feature extraction [36], dense denotes the fully connected layer, and RELU is the activation function that captures the key to nonlinear relationships.

We then adopted the idea of an information bottleneck to reduce feature dimensionality. Specifically, the goal of the idea is to compress the input-redundant features R to obtain the potential variable P that can maximally represent its category C, that is, minimize the mutual information M(R,P) and maximize the mutual information M(C,P) [49]. Based on the information bottleneck theory, the above process can be regarded as a maximization problem with the following formula:

$$\underset{p({p}\mid{r})}{max}\;M(C,P)-{\lambda}{M}(R,P)$$

where $\lambda$ is the Lagrangian quantity and M denotes the mutual information operation between two variables. The mutual information is calculated as follows:

$$\text{M}\left(\text{R},\text{P}\right)=\int \text{q}\left(\text{r},\text{p}\right)\left(\text{log q}\left(\text{r},\text{p}\right)-\text{log q}\left(\text{r}\right)-\text{log q}\left(\text{p}\right)\right)dr\ dp$$

Because q(c|p) and q(p) are not computable and the Kullback–Leibler scatter is positive, the above equation can be approximated using a variational approximation as follows:

$$\text{KL}\left[\text{q}\left(\text{c}|\text{p}\right),\text{t}\left(\text{c}|\text{p}\right)\right]\ge 0$$

$$\int q\left(c|p\right)\text{log}\;q\left(c|p\right)\;dc\ge \int q\left(c|p\right)\;\text{log}\;t\left(c|p\right)\;dc$$

Thus, M(C,P) can be approximated as follows:

$$\begin{aligned}\mathrm M\left(\mathrm C,\mathrm P\right) &\geq\int\mathrm q\left(\mathrm c,\;\mathrm p\right)\left(\log\left(\mathrm c\vert\mathrm p\right)-\log\;\mathrm q\left(\mathrm c\right)\right)\;dc\mathit\;dp\;\\ &=\int\mathrm q\left(\mathrm c,\mathrm p\right)\;\log\;\mathrm t\left(\mathrm c\vert\mathrm p\right)\;dc\mathit\;dp-\int\mathrm q\left(\mathrm c\right)\;\log\;\mathrm q\left(\mathrm c\right)\;dc\\ &=\int\mathrm q\left(\mathrm c,\;\mathrm p\right)\;\log\;\mathrm t\left(\mathrm c\vert\mathrm p\right)\mathit\;dc\mathit\;dp+\mathrm G\left(\mathrm C\right)\end{aligned}$$

where G(C) is irrelevant to the optimization objective and can be ignored. Furthermore, M(C,P) can be expressed using the Markov chain and edge probability density formula as follows:

$$\text{M}\left(\text{C},\text{P}\right)\ge \int \text{q}\left(\text{r}\right)\text{q}\left(\text{c}|\text{r}\right)\text{q}\left(\text{p}|\text{r}\right)\, \text{log t}\left(\text{c}|\text{p}\right)dr\ dc\ dp$$

Similarly, M(R,P) can be expressed as follows:

$$\text{M}\left(\text{R},\text{P}\right)\le \int \text{q}\left(\text{r}\right)\text{q}\left(\text{p}|\text{r}\right)\left(\text{log q}\left(\text{p}|\text{r}\right)-\text{s}\left(\text{p}\right)\right)dr\,dp$$

Combining the boundaries of M(C,P) and M(R,P) can be expressed as follows:

$$M(C,P)-{\lambda}{M}(R,P)\geq{L}$$

L is the lower bound, and the optimization objective can be approximated as follows:

$$\mathrm L\approx\frac{1}{\mathrm K}\sum_{\mathrm i=1}^{\mathrm K}\left[dz\;\mathrm q(p\vert r_i)\log\;\mathrm t\left({\mathrm c}_{\mathrm i}\vert\mathrm p\right)-\lambda q\left(\mathrm p\vert{\mathrm r}_{\mathrm i}\right)\log\frac{\mathrm q\left(\mathrm p\vert{\mathrm r}_{\mathrm i}\right)}{\mathrm s\left(\mathrm p\right)}\right]$$

We estimated the gradient of the lower bound L using the reparameterization technique and calculated it as follows:

$$z=E\left(r\right)+\varepsilon D\left(r\right)$$

where E(r) is the mean of r, D(r) is the variance of r, and is an auxiliary noise variable and follows a standard normal distribution.

Because the VFC is random in terms of weight initialization and sampling, the feature set processed by the algorithm exhibits a certain degree of randomness. Therefore, we simultaneously generated multiple features with the same dimensions. After several rounds of validation, the feature set with the highest MCC value and the smallest possible number of feature dimensions was the final feature set. Finally, we obtained a final feature set containing 15 features.

### Classification method

Since Chen first proposed the XGBoost algorithm in 2017 [50], it has received considerable attention as an integrated learning algorithm based on gradient enhancement [51–54]. XGBoost accumulates the predictions of the k-tree by weighting at each iteration step and uses the information of the first-order derivative from the loss function to adjust the model and optimize the final prediction of the model using the following formula:

$$\upphi \left({\text{x}}_{\textrm{i}}\right)=\sum_{\text{k}=1}^{\textrm{K}}{\text{f}}_{\textrm{k}}\left({\text{x}}_{\textrm{i}}\right)=\sum_{\text{k}=1}^{\textrm{K}}{\upomega }_{{\text{q}}_{\textrm{k}}\left({\text{x}}_{\textrm{i}}\right)}$$

where ${q}_{k}\left({x}_{i}\right)$ is the prediction model for the kth tree, w is the leaf weight, ${x}_{i}$ is the characteristics of the sample, and *y* is the algorithm’s prediction result. Given an input, it will be determined the leaf node to which it belongs is based on the branching condition (denoted as q) of the tree structure species. The prediction is obtained by accumulating the scores (denoted as w) of all the leaf nodes through which this input passes. For the algorithm to learn the sample information better, the objective function can be expressed as follows:

$$\mathrm{obj}\left(\mathrm\theta\right)=\sum_{\mathrm i=1}^{\mathrm n}\ell\left({\mathrm y}_{\mathrm i},{\widehat{\mathrm y}}_1\right)+\sum_{\mathrm k=1}^{\mathrm K}\Omega\left({\mathrm t}_{\mathrm k}\right)=\sum_{\mathrm i=1}^{\mathrm n}\ell\left({\mathrm y}_{\mathrm i},{\widehat{\mathrm y}}_1\right)+\gamma T+\frac{1}{2}\mathrm\lambda\parallel\mathrm\omega\parallel^2$$

where ${\ell}$ is used to measure the difference between the algorithm’s prediction and the actual labels. *Ω* is the regularization term, consisting of the leaf node count and leaf weights. The parameter in the regularization term controls leaf number, and the parameter controls leaf weights.

### Evaluation metrics

The k-fold cross-validation assessment model was previously found to yield more objective assessment results [55–59] and was used in this study as well. This method splits the dataset equally into k-folds, selecting onefold at a time as the validation set and the rest of the folds as the training set, and repeating this k times. The averages of the evaluations were used as the cross-validation results. To demonstrate that the model performs better on both positive and negative samples, we used the following relevant formulas as evaluation metrics:

$$ACC=\frac{TN+TP}{TN+FP+FN+TP}$$

$$SN=\frac{TP}{TP+FN}$$

$$SP=\frac{TN}{TN+FP}$$

$$\text{MCC}=\frac{\text{TN}\times \text{TP}-\text{FN}\times \text{FP}}{\sqrt{(\textrm{TN}+\textrm{FP})\times (\textrm{FN}+\textrm{TP})\times (\textrm{TN}+\textrm{FN})\times (\textrm{TP}+\textrm{FP})}}$$

$${F1}_{score}=\frac{2TP}{2TP+FP+FN}$$

where TP represents the amount of data categorizing antioxidant proteins as antioxidant proteins, FP represents the amount of data categorizing nonantioxidant proteins as antioxidant proteins, TN represents the amount of data categorizing nonantioxidant proteins as nonantioxidant proteins, and FN represents the amount of data categorizing antioxidant proteins as nonantioxidant proteins. The Matthews correlation coefficient (MCC) represents the ability of the model to balance the predictive accuracy of positive and negative samples [48].

## Results and discussion

### Algorithm overview

**Fig. 2:**
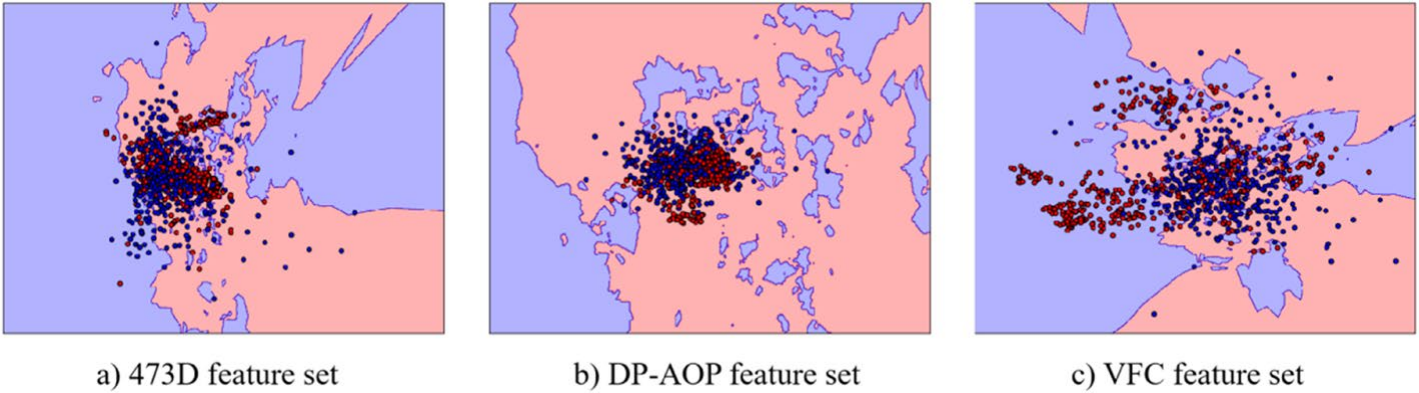
Sample distribution visualization of the feature set obtained based on different algorithms. **a** Sample distribution visualization of the feature set using the original 473D feature set, **b** sample distribution visualization of the 17-dimensional enlistment used in DP-AOP, and **c** sample distribution visualization of the 15-dimensional enlistment obtained based on the dimensionality reduction of the VFC features. Red dots represent positive samples, and blue dots represent negative samples in the figure

To assess the performance of the dimensionality reduction algorithm, we first plotted sample distributions before and after dimensionality reduction. Owing to the clear description of feature contributions in DP-AOPs, we refer to the table provided and visualize these features for comparison. For a fair assessment, we chose the same number of features as the number of feature dimensions we adopted, i.e., the first 15 features, based on the contribution of the features in the DP-AOPs table from highest to lowest. Data points before dimensionality reduction were found to be highly interconnected (Fig. 2a), with the red and blue data points appearing particularly overlapping each other in some areas. In addition, the decision boundary was found to be complex and irregular, hindering the capture of useful information. In Fig. 2b, the classification boundaries between the red and blue data points are relatively more clear, with several regions densely populated with a single category, yet some regions appeared to contain both types. Upon dimensionality reduction, the separation between the red and blue data points in some regions was improved compared to the original features and DP-AOPs. In addition, the decision boundary line separated the two types of data points. In summary, we conclude that the low-dimensional features obtained by the VFC dimensionality reduction method enable a more generalized and efficient classification.

Although the main idea behind VFC feature dimensionality reduction is to transform and combine the original features nonlinearly in the latent space, the method still has a preference for certain features. Thus, to some extent, certain features and their potential relationships are more important to identify antioxidant proteins. In addition, the source of the features after dimensionality reduction may reveal their biological significance. We determined the importance of the original features and the relationship between the features before and after dimensionality reduction using the SHAP method. First, the SHAP values between the original feature vector and 15-dimensional feature vector were calculated for all samples. The SHAP values of all samples were then averaged to obtain absolute values. To obtain the main components of the dimensionality reduction feature, we sorted the SHAP values of the 473-dimensional features corresponding to each dimensionality reduction feature in descending order, and thus obtained the features in descending order of importance. Considering the large number of features, we chose features with SHAP values greater than 0.012 as references.

**Fig. 3:**
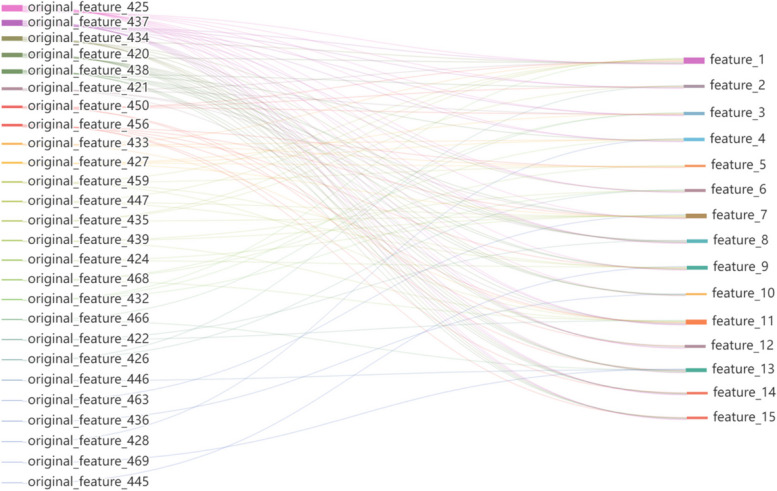
Visualization of the relationship between the original and dimensionality reduction feature vectors obtained based on SHAP values. The original features on the left side of the figure are arranged in descending order based on SHAP values, and the features on the right side are arranged in the order of feature names

The relationship between the original feature vector obtained based on the SHAP value and the dimensionality-reduction feature vector is shown in Fig. 3. The original features on the left side of the graph are listed in decreasing order of SHAP values, and the features on the right side are listed in order of feature name. Ten features were based on the structure probability matrix, and 26 features were based on the consensus sequence, as shown in the figure. Additionally, the five features with the largest SHAP values were based on a consensus sequence. Here, consensus sequences were found to play an essential role in predicting antioxidant proteins.

### Performance evaluation based on k-fold cross-validation

To estimate the model performance more reliably, the k-fold cross-validation method was used to estimate model performance. Specifically, the antioxidant dataset was split into five equally sized subsets, and a different subset was selected as test data, whereas the remaining four were used for model training each time model performance was evaluated. This process was repeated five times. This cross-validation process reduces accidental errors by repeating it many times for different combinations of subsets, ultimately yielding more objective model evaluation results. For performance evaluation, a benchmark dataset was constructed using the dataset developed by Feng et al. (2016). Table 1 presents the performance results of the developed classifier on this dataset for five instances after fivefold cross-validation, as well as the average performance of the five instances. The results of the experiments objectively demonstrated the accuracy and generalization ability of the model, as well as its ability to classify antioxidant proteins.

**Table 1:** Performance results and average performance results of the fivefold cross-validation method based on the benchmark dataset

Times	ACC	SN	SP	MCC	F1
1	96.62	96.44	96.80	93.24	96.60
2	96.78	96.30	97.22	93.55	96.62
3	95.49	95.25	95.74	90.98	95.56
4	95.65	93.31	98.29	91.44	95.79
5	94.84	95.68	94.04	89.69	94.74
Average	95.88	95.40	96.42	91.78	95.86

### Comparison of Rore classifier performance with those of other classifiers

**Fig. 4:**
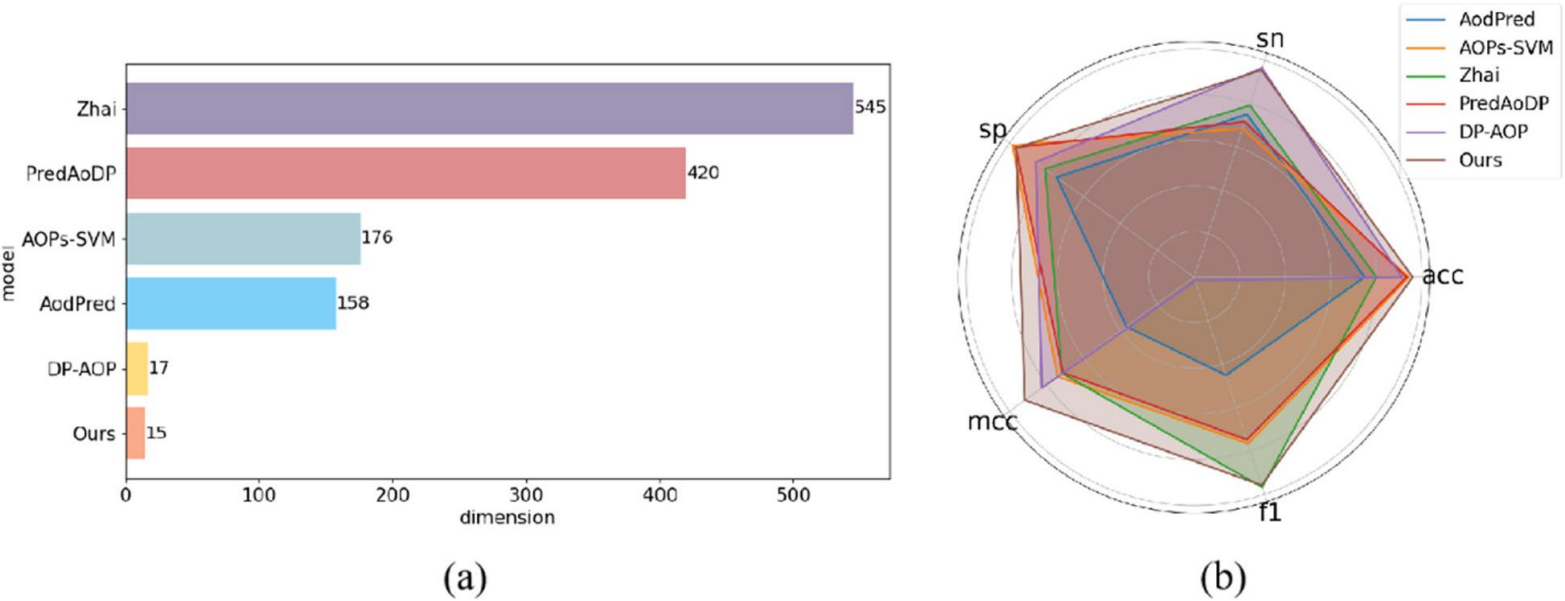
Comparison of Rore’s performance with other models. **a** Comparison based on the dimension of the feature vectors used. **b** Comparison based on SN, SP, MCC, ACC, and F1 evaluation metrics

We adopted the benchmark dataset used in the k-fold cross-validation method and tested the performances of the Rore model and the other five methods. As shown in Fig. 2, Fig. 4, and Table 2, Rore model outperforms all the other methods owing largely to retaining of the original information and discarding of noise. In clinical applications, the advantage in terms of the MCC values is significant, indicating that the classifier performs well on unbalanced datasets. In practice, more importance is given to MCC and ACC values. Therefore, we sacrificed SN, SP, and F1 scores within acceptable limits.

**Table 2:** Performance comparison of Rore with other classifiers

Model	ACC	SN	SP	MCC	F1	Dimension
AodPred	74.79	75.09	74.48	36.8	45.2	158
Zhai	80	79.2	80.8	71.65	**96.77**	545
DP-AOP	91.08	**96.4**	85.8	82.6	91.5	17
PredAoDP	93.18	71.65	96.77	71.2	74.9	420
AOPs-SVM	94.2	68.5	**98.5**	74.1	76.7	176
Rore	**95.88**	95.4	96.42	**91.78**	95.86	**15**

## Conclusion

To overcome the limitations of feature selection in existing protein identification methods, a classifier based on feature dimensionality reduction was proposed in this study. By mapping the original features into the potential space to obtain a compressed representation with all feature information, the problem of information loss caused by the previous use of feature selection is solved. Experimental results show that on the benchmark dataset, our classifier model outperforms other existing classifiers in three metrics, MCC, F1, and ACC, using only 15-dimensional features. A high MCC value indicates superiority when dealing with unbalanced datasets. It shows great potential in clinical applications. Therefore, we can conclude that the Rore classifier can obtain more robust features to achieve superior recognition ability. Given the excellent robustness of the Rore classifier, its application potential in other recognition areas may be investigated in future studies.

## Data Availability

Data is provided within the manuscript or supplementary information files.
